# Editorial for This Special Issue on Energy Conversion Materials and Devices and Their Applications

**DOI:** 10.3390/mi16080943

**Published:** 2025-08-17

**Authors:** Bin Liu, Yaling Wang, Lei Liu

**Affiliations:** State Key Laboratory of Coal and CBM Co-Mining, School of Energy and Power Engineering, North University of China, Taiyuan 030051, China

## 1. Introduction

The global push toward sustainable energy, driven by soaring energy demands, escalating environmental concerns, and urgent climate challenges, has catalyzed remarkable advancements in energy conversion materials and devices [1,2,3,4,5]. This necessitates next-generation systems that prioritize higher efficiency, reduced toxicity, enhanced stability, and sustainable material bases [6,7,8,9,10]. Breakthroughs in materials science and device engineering are now paving the way toward resilient and intelligent energy solutions [11,12,13,14]. This Special Issue presents cutting-edge research addressing these critical needs, exploring innovations spanning from perovskite solar cells, solid-state lithium-metal batteries, and sodium-ion batteries to electrochromic devices, organic light-emitting diodes (OLEDs), and thermally activated light emitters.

This Special Issue includes seven original research articles and three review articles. These works not only reflect the latest scientific innovations but also emphasize application-orientated strategies with potential for commercial translation. In this editorial, we will provide an integrated overview of the thematic developments within this Special Issue and highlight the broader significance of each contribution.

Energy storage lies at the heart of the clean energy transition [15,16,17,18]. Batteries are indispensable for grid stabilization, electric vehicles, and portable electronics. However, conventional lithium-ion batteries face bottlenecks in resource availability, safety, and energy density [19,20,21,22]. In this Special Issue, Li et al. [23] report on ZnMn_2_O_4_/V_2_CT_x_ composites via high-temperature calcination to synergize spinel-type oxides with MXene to enhance lithium storage. The unique lamellar rod-like structure achieves an improved specific discharge capacity (163 mAh g^−1^) and superior cycle life compared to pure ZnMn_2_O_4_. The synergistic interaction between ZnMn_2_O_4_ and V_2_CT_x_ is key to the composite’s remarkable performance, significantly boosting its lithium-ion storage capacity. This demonstrates how composite architectures can enhance electrochemical kinetics and mitigate volume expansion during charge/discharge cycles.

Advancing toward the next generation of lithium-ion batteries, Lee et al. [24] comprehensively review strategies to stabilize lithium metal anodes in solid-state electrolytes (SSEs), a critical step in enabling the production of all-solid-state lithium-ion batteries. While lithium metal offers unparalleled energy density, its high reactivity and dendritic growth pose serious challenges. The authors systematically compare sulfide-, oxide-, and polymer-based SSEs, evaluating their electrochemical performance, ion conductivity, and interfacial compatibility. Sulfide SSEs, though promising due to their high conductivity, are susceptible to dendrite formation and suffer from a limited electrochemical window. In contrast, oxide SSEs (e.g., Li_7_La_3_Zr_2_O_12_) offer superior chemical stability but suffer from high interfacial resistance due to Li_2_CO_3_ formation. Polymer electrolytes provide flexibility but lack sufficient ion mobility.

Another dimension involves strategies to stabilize lithium metal anodes in SSEs [25,26,27]. Ahangari et al. [28] introduce a surface engineering approach using ultrathin Al_2_O_3_ coatings (via atomic layer deposition) to resolve structural instability and parasitic reactions in Ni-rich Li(Ni_0.8_Co_0.1_Mn_0.1_)O_2_ cathodes. The modified Ni-rich Li(Ni_0.8_Co_0.1_Mn_0.1_)O_2_ exhibits capacity retention improvements of 5%, 9.11%, and 11.28% compared to the pristine material at 4.3 V, 4.4 V, and 4.5 V, respectively. This enhances cycling stability and capacity retention, offering insights into interfacial engineering for high-capacity cathodes.

For grid-scale storage, sodium-ion systems are emerging as cost-effective alternatives [29,30]. In this Special Issue, Ahangari et al. [31] comprehensively review layered transition metal oxide cathodes for sodium-ion batteries. Their analysis focuses on structural properties, electrochemical performance, degradation mechanisms, and the intrinsic and extrinsic factors underlying instability. Furthermore, they highlight advances in doping, surface modification, and composites aimed at mitigating capacity fading and structural instability, suggesting promising prospects for this technology.

Electrochromic materials represent another cornerstone of energy-conversion applications, especially in smart windows and adaptive optics [32,33,34]. Two studies in this Special Issue exemplify breakthroughs in tungsten oxide (WO_3_)-based electrochromic films, offering transformative insights for future applications. Zhang et al. [35] utilize ex situ spectroscopic ellipsometry to investigate Li^+^ insertion mechanics in WO_3_ films, revealing a layer-by-layer permeation process critical in optical modulation. Their findings provide insights into optical modulation for zoom lenses and phase modulators. Wang et al. [36] advance this further with an MXene-regulated, ethylene glycol-induced coral-like TiO_2_@WO_3_ heterostructure, achieving a coloring efficiency of 137.02 cm^2^C^−1^ and >90% performance retention after 4000 cycles. Together, these works demonstrate how nanoscale design can unlock new paradigms in dynamic optics and energy efficiency.

Solar energy remains vital in global decarbonization strategies, but lead toxicity and stability concerns hinder the mass adoption of perovskite solar cells [37,38,39]. In this Special Issue, Gao et al. [40] tackle this issue by developing compact (CH_3_NH_3_)_3_Bi_2_I_9_ perovskite films using a two-step vapor-assisted method. Their approach yields morphologically optimized films with enhanced power conversion efficiency (~1.13%) and device stability, retaining 99% performance after 60 days in the glove box environments. This suggests that with refined crystallization control and morphological optimization, bismuth-based perovskites could play a pivotal role in non-toxic photovoltaics. This work paves the way for new classes of high-performance, lead-free, solution-processable solar materials, especially in regions with environmental regulations in place regarding heavy metals.

At the energy–optoelectronics frontier, light-emitting technologies are becoming integral to energy-efficient devices [41,42]. In this Special Issue, Zhang et al. [43] review host-free white OLEDs using thermally activated delayed fluorescence (TADF). This technology enables up to 100% internal quantum efficiency and simplifies device architecture, paving the way for next-generation flexible and efficient displays and lighting systems. By comparing TADF, TADF phosphorescence, and all-TADF materials, the authors discuss the benefits and current challenges in this area.

For telecommunications, optical amplifiers must cover the full NIR window (1000–1600 nm) [44,45,46]. Traditional rare-earth-doped amplifiers (e.g., erbium) are limited by narrow emission bands. In this Special Issue, Song and Zhang [47] demonstrate that Bi/Cr co-doped aluminosilicate glasses can achieve broadband emissions (650–1600 nm), surpassing traditional rare-earth-doped amplifiers and enhancing bandwidth for fiber-optic networks.

The future of energy hinges not only on performance but also on scalability, safety, accessibility, and environmental impact. The studies in this Special Issue converge on a common vision: material innovation that is informed by application-specific needs. However, challenges remain. Bridging the lab-to-fab gap, ensuring material reproducibility, and integrating these devices into complex systems require interdisciplinary efforts. We hope that this Special Issue will serve as both a snapshot of current progress and a springboard for future breakthroughs.

We extend our deepest gratitude to all the authors for their contributions to this Special Issue. We also sincerely appreciate the reviewers for their time and dedication in ensuring the quality of this publication. Finally, we are grateful to the staff of the *Micromachines* Editorial Office, particularly Mr. Lebron Tu, for their invaluable assistance.
